# Peroxiredoxin 2 Is a Potential Objective Indicator for Severity and the Clinical Status of Subarachnoid Hemorrhage Patients

**DOI:** 10.1155/2023/5781180

**Published:** 2023-02-06

**Authors:** Zheng Peng, Cong Pang, Xiao-Jian Li, Hua-Sheng Zhang, Jia-Tong Zhang, Qi Zhu, Hua-Jie Xu, Yong-Yue Gao, Zong Zhuang, Wei Li, Qing-Rong Zhang, Yue Lu, Chun-Hua Hang

**Affiliations:** ^1^Department of Neurosurgery, Affiliated Nanjing Drum Tower Hospital, Medical School, Nanjing University, Nanjing, China; ^2^Department of Neurosurgery, Nanjing Drum Tower Hospital Clinical College of Nanjing Medical University, Nanjing, 210008 Jiangsu, China

## Abstract

**Purpose:**

We have demonstrated that peroxiredoxin 2 (Prx2) released from lytic erythrocytes and damaged neurons into the subarachnoid space could activate microglia and then result in neuronal apoptosis. In this study, we tested the possibility of using Prx2 as an objective indicator for severity of the subarachnoid hemorrhage (SAH) and the clinical status of the patient.

**Materials and Methods:**

SAH patients were prospectively enrolled and followed up for 3 months. Cerebrospinal fluid (CSF) and blood samples were collected 0-3 and 5-7 days after SAH onset. The levels of Prx2 in the CSF and the blood were measured by an enzyme-linked immunosorbent assay (ELISA). We used Spearman's rank coefficient to assess the correlation between Prx2 and the clinical scores. Receiver operating characteristic (ROC) curves were used for Prx2 levels to predict the outcome of SAH by calculating the area under the curve (AUC). Unpaired Student's *t*-test was used to analyze the differences in continuous variables across cohorts.

**Results:**

Prx2 levels in the CSF increased after onset while those in the blood decreased. Existing data showed that Prx2 levels within 3 days in the CSF after SAH were positively correlated with the Hunt-Hess score (*R* = 0.761, *P* < 0.001). Patients with CVS had higher levels of Prx2 in their CSF within 5-7 days after onset. Prx2 levels in the CSF within 5-7 days can be used as a predictor of prognosis. The ratio of Prx2 in the CSF and the blood within 3 days of onset was positively correlated with the Hunt-Hess score and negatively correlated with Glasgow Outcome Scale (GOS; *R* = ‐0.605, *P* < 0.05).

**Conclusion:**

We found that the levels of Prx2 in the CSF and the ratio of Prx2 in the CSF and the blood within 3 days of onset can be used as a biomarker to detect the severity of the disease and the clinical status of the patient.

## 1. Introduction

The most common cause of spontaneous subarachnoid hemorrhage (SAH) is a ruptured intracranial aneurysm, accounting for approximately 85% of all SAH cases, with vascular malformations and vasculitis making up the rest [1]. SAH accounts for 5-10% of all stroke-related diseases. Compared with other types of strokes, SAH patients are younger and bear a heavier mental loss and economic burden to their families and society [2, 3]. Although neurosurgical clipping or endovascular embolization can successfully remove the aneurysm, complications such as cerebral vasospasm (CVS), hydrocephalus, and delayed cerebral ischemia (DCI) still lead to a poor prognosis in SAH patients [4]. An assessment of SAH clinical status often lags disease progression. Therefore, it is particularly important to find new methods for effective early identification of SAH patients' clinical status.

Peroxiredoxin (Prx) family is a class of peroxidase proteins that can scavenge peroxides and inhibit apoptosis [5, 6]. Prx2 has been reported to be one of the most elevated proteins in the cerebrospinal fluid (CSF) of SAH patients [7]. Our previous work showed that Prx2 was mainly expressed in the neurons. After SAH, Prx2 was significantly increased. Intracellular Prx2 inhibited neuronal apoptosis and protected the neurons from oxidative stress damage [8]. Lytic erythrocytes and damaged neurons can release Prx2. Thus, extracellular Prx2 activates microglia and promotes neuronal apoptosis [9, 10]. In this study, we analyzed the content of Prx2 in the CSF and the blood of SAH patients by an enzyme-linked immunosorbent assay (ELISA) and found that Prx2 can be used as a biomarker for judging the clinical status of the patients. This finding provides a new reference idea for the early identification of the clinical conditions of SAH patients.

## 2. Materials and Methods

### 2.1. Clinical Ethics

The collection and use of the clinical samples were approved by the Ethics Committee of Nanjing Drum Tower Hospital (No. 2022-294-01), and informed consent was obtained from the patients.

### 2.2. Case Enrollment and Sample Collection

The inclusion criteria for the experimental group were as follows: (1) older than 18 years, (2) all the subjects met the diagnostic criteria for SAH and sought medical attention within 24 h of onset, and (3) computed tomography angiography/digital subtraction angiography (CTA/DSA) excluded cerebrovascular diseases such as arteriovenous malformation, arteriovenous fistula, and moyamoya disease. Participants in the control group had no central nervous system (CNS) disease but required a lumbar puncture. All the blood and CSF samples were centrifuged (3000 r/min, 10 mins) immediately after collection and placed at -80°C. Brain tissue samples from the SAH patients were stored in formaldehyde for subsequent immunofluorescence (IF) detection. Thirty-four patients were enrolled in this study, of whom 20 obtained CSF within 3 days, 28 obtained CSF and the blood within 5-7 days, and 14 obtained the blood within 3 days. The patient's characteristic is shown in Table 1.

### 2.3. ELISA Test

The levels of Prx2 in the CSF and the blood were detected using an ELISA kit (MBS2510631, MyBioSource). Following the steps in the instructions briefly, we added the standard working solution or the samples to the wells, sealed them, and incubated them for 90 mins at 37°C. After removing the liquid, we added biotinylated detection Ag working solution to each well and incubated for 60 mins at 37°C. After washing three times, we added the HRP conjugate working solution to each well and incubated it for 30 mins at 37°C. Then, the substrate reagent was added to each well. After incubating for about 15 mins at 37°C, stop solution was added, and the optical density (OD) value of each well was determined at once with a microplate reader set to 450 nm. We calculated Prx2 levels from the standard curve.

### 2.4. Severity Assessment of the Disease

The admission Hunt-Hess score was used to evaluate the severity of the patient's disease, and the score was evaluated by the experienced clinicians using a single-person assessment to ensure the consistency of the score.

### 2.5. Evaluation of CVS

The CVS criteria we used were as follows: (1) after treatment, the symptoms of the SAH patients were significantly relieved and then progressively worsened, accompanied by symptoms such as high fever; (2) the disorders of consciousness progressively worsened; (3) the patient had neurological deficits, such as limb dysfunction and sensory impairment; (4) the patient had symptoms of increased intracranial pressure; (5) DSA showed the presence of CVS; and (6) transcranial Doppler ultrasonography showed a change in the blood flow velocity and the arterial parameters [11]. After excluding secondary hemorrhage, hematoma caused by hemorrhage, hydrocephalus, and other causes, two or more occurrences were diagnosed as CVS.

### 2.6. Prognostic Assessment

Three months after the patients were discharged from the hospital, we conducted a telephone follow-up visit and assessed the GOS, 1-3 as poor prognosis and 4-5 as good prognosis [12]. The GOS was assessed by an experienced clinician using a single-person assessment to ensure consistency of the score.

### 2.7. IF Test

The IF assays were tested as mentioned in our previous article [13]. Frozen sections of the patient's brain tissue were rewarmed. The cell membranes were permeabilized with 3% Triton X-100 followed by blocking with a blocking solution. The sections were incubated with the primary antibody overnight at 4°C, followed by the secondary antibody incubation at room temperature. Nuclei were stained with DAPI (P0131, Beyotime) containing an antiquencher, and then, the sections were photographed using a fluorescence microscope. The primary antibodies used were anti-Prx2 (10545-2-AP, Proteintech, 1 : 200), anti-NeuN (66836-1-Ig, Proteintech, 1 : 200), anti-Iba-1 (66827-1-Ig, Proteintech, 1 : 200), and anti-GFAP (60190-1-Ig, Proteintech, 1 : 200). The secondary antibodies used were CoraLite594-conjugated donkey anti-rabbit IgG (SA00013-8, Proteintech, 1 : 400) and CoraLite488-conjugated donkey anti-mouse IgG (SA00013-5, Proteintech, 1 : 400).

### 2.8. Statistical Analysis

SPSS 27 (IBM SPSS Statistics, USA) and GraphPad Prism 9.0 for Windows (GraphPad Software, USA) were used for the data analysis. Unpaired Student's *t*-test was used to analyze the differences in continuous variables across cohorts. Data were expressed as mean ± standard deviation (SD), and *P* < 0.05 was considered statistically significant. We used Spearman's rank coefficient to assess the correlation between Prx2 and the clinical scores. Receiver operating characteristic (ROC) curves were used for Prx2 levels to predict the outcome of SAH by calculating the area under the curve (AUC).

## 3. Results

### 3.1. Localization of Prx2 in the Patient Brain Tissue

**Figure 1:**
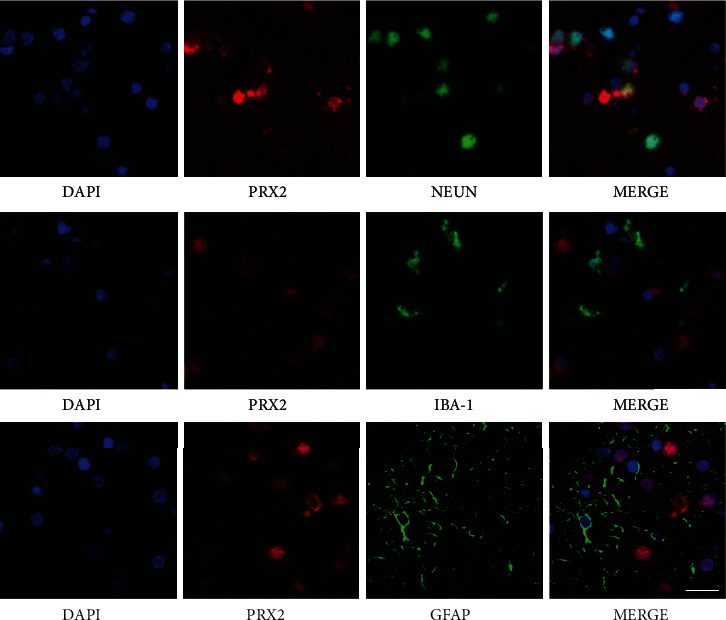
Localization of Prx2 in the patient's brain tissue. We explored the localization of Prx2 in the brain tissue using IF (NeuN: neuronal marker, Iba-1: microglia marker, GFAP: astrocyte marker, scale bar: 20 *μ*m). IF: immunofluorescence.

As shown in Figure 1, IF showed that Prx2 was mainly localized to neurons rather than microglia or astrocytes. In our previous study, we explored the localization of Prx2 in the mouse brain tissue. The two reports are consistent, and Prx2 was also localized in the neurons of the mice [8].

### 3.2. Expression Patterns of Prx2 after the Onset

**Figure 2:**
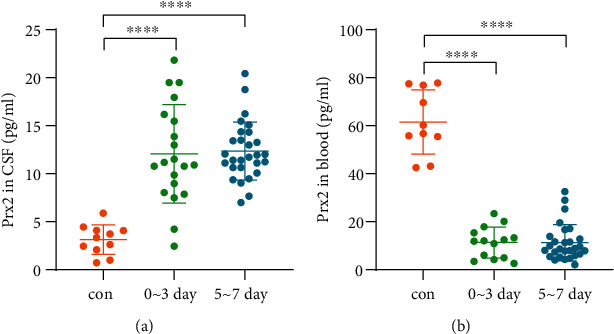
Expression patterns of Prx2 after SAH onset. (a) Changes in Prx2 levels in CSF after SAH onset (^∗∗∗∗^*P* < 0.0001). (b) Changes in Prx2 levels in the blood after SAH onset (^∗∗∗∗^*P* < 0.0001).

We explored the trend of Prx2 expression as shown in Figure 2. After onset, the levels of Prx2 in the CSF continued to increase, while those in the blood decreased with statistical significance in the acute phase (0-3 days, *P* < 0.0001) and the subacute phase (5-7 days, *P* < 0.0001). There was no statistical difference in the Prx2 levels in the CSF and the blood between acute and subacute phases.

### 3.3. Relationship between Prx2 Levels and Disease Severity

**Figure 3:**
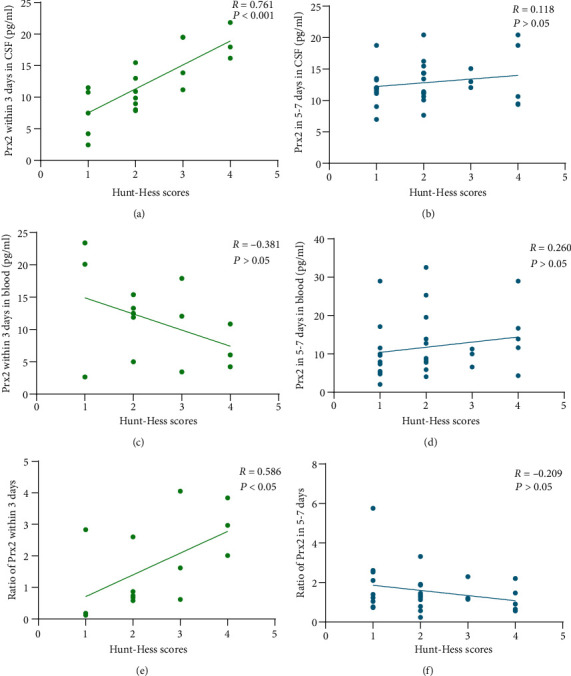
Correlation between the Prx2 level and the Hunt-Hess scores after SAH onset. (a) Correlation between Prx2 levels in CSF within 3 days and Hunt-Hess scores after the onset. (b) Correlation between Prx2 levels in CSF within 5-7 days and Hunt-Hess scores after the onset. (c) Correlation between Prx2 levels in the blood within 3 days and Hunt-Hess scores after the onset. (d) Correlation between Prx2 levels in the blood within 5-7 days and Hunt-Hess scores after the onset. (e) Correlation between the ratio of Prx2 within 3 days and Hunt-Hess scores after the onset. (f) Correlation between the ratio of Prx2 in 5-7 days and Hunt-Hess scores after the onset.

We assessed the correlation of the Prx2 levels in the blood and CSF with the Hunt-Hess score at different times. The levels of Prx2 in the CSF within 3 days of onset were positively correlated with the Hunt-Hess scores (*R* = 0.761, *P* < 0.001, Figure 3(a)). There was no correlation between Prx2 levels in the CSF within 5-7 days and Hunt-Hess scores (Figure 3(b)). There was no correlation between Prx2 levels in the blood and Hunt-Hess score within 3 days and 5-7 days of onset (Figures 3(c) and 3(d)).

### 3.4. Relationship between Prx2 Levels and CVS

**Figure 4:**
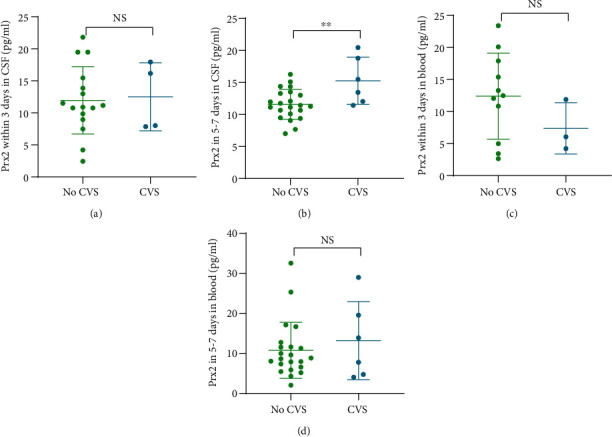
Relationship between Prx2 levels and CVS after SAH onset. (a) Differences in Prx2 levels in CSF within 3 days after the onset in patients with and without CVS (NS: no significance). (b) Differences of Prx2 levels in CSF in 5-7 days after the onset in patients with and without CVS (^∗∗^*P* < 0.01). (c) Differences in Prx2 levels in the blood within 3 days after the onset in patients with and without CVS (NS: no significance). (d) Differences of Prx2 levels in the blood within 5-7 days after the onset in patients with and without CVS (NS: no significance).

CVS occurred in 7 out of the 34 patients during hospitalization. We compared the differences between the Prx2 levels in the CSF and the blood in patients with and without CVS, as shown in Figure 4. It was found that the levels of Prx2 in the CSF of the patients with CVS were higher within 5-7 days (*P* < 0.01), although the levels of Prx2 in the blood within 0-3 days and 5-7 days were not different between the two groups.

### 3.5. Relationship between Prx2 and Prognosis

**Figure 5:**
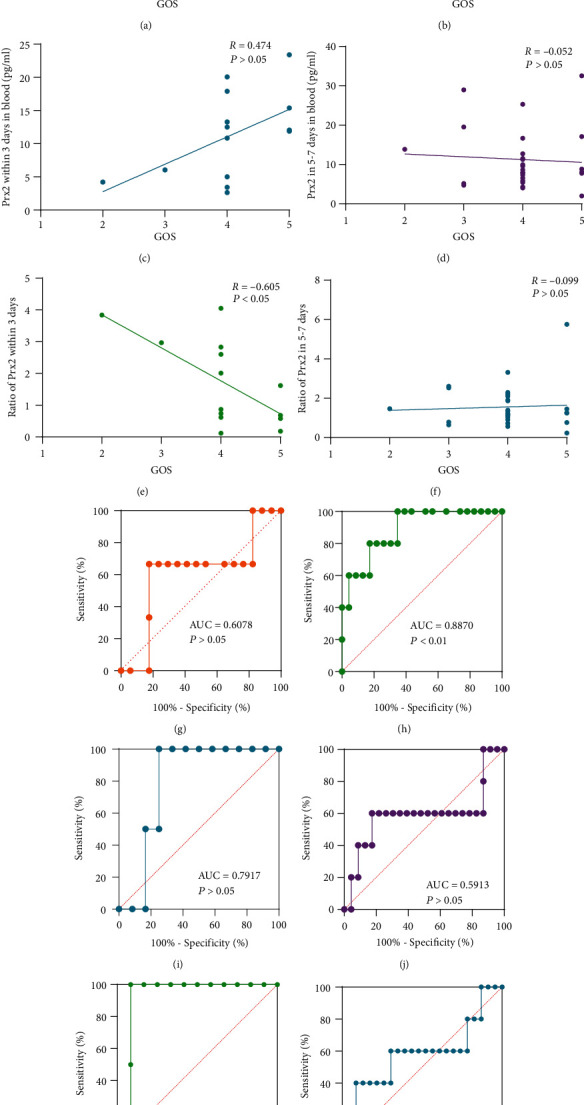
Relationship between Prx2 levels and the outcome of SAH. (a) Correlation between Prx2 levels in CSF within 3 days and the GOS after discharge. (b) Correlation between Prx2 levels in CSF within 5-7 days and the GOS after discharge. (c) Correlation between Prx2 levels in the blood within 3 days and the GOS after discharge. (d) Correlation between Prx2 levels in the blood within 5-7 days and the GOS after discharge. (e) Correlation between the ratio of Prx2 within 3 days and the GOS. (f) Correlation between the ratio of Prx2 within 5-7 days and the GOS. (g) ROC curves for Prx2 levels within 3 days in CSF to predict poor outcome of SAH (AUC = 0.6078; *P* > 0.05; 95% confidence interval, 0.2279 to 0.9878). (h) ROC curves for Prx2 levels in 5-7 days in CSF to predict poor outcome of SAH (AUC = 0.8870; *P* < 0.01; 95% confidence interval, 0.7436 to 1.000). (i) ROC curves for Prx2 levels within 3 days in the blood to predict poor outcome of SAH (AUC = 0.7917; *P* > 0.05; 95% confidence interval, 0.5625 to 1.000). (j) ROC curves for Prx2 levels in 5-7 days in the blood to predict poor outcome of SAH (AUC = 0.5913; *P* > 0.05; 95% confidence interval, 0.2453 to 0.9373). (k) ROC curves for the ratio of Prx2 within 3 days to predict poor outcome of SAH (AUC = 0.9167; *P* > 0.05; 95% confidence interval, 0.7603 to 1.000). (l) ROC curves for the ratio of Prx2 within 5-7 days to predict poor outcome of SAH (AUC = 0.5739; *P* > 0.05; 95% confidence interval, 0.2573 to 0.8905).

As shown in Figures 5(a)–5(d), we evaluated the correlation between the Prx2 levels and GOS three months after the discharge, and only Prx2 levels in the CSF on days 5-7 of onset were correlated with the GOS (*R* = ‐0.487, *P* < 0.01). We used ROC curves to assess the extent to which Prx2 can predict a poor prognosis. The AUCs for Prx2 levels in the CSF and the blood at 0-3 days, and 5-7 days, as predictors for poor outcome, were 0.6078, 0.8870, 0.7917, and 0.5913, respectively, and only CSF at 5-7 days had statistical significance (*P* < 0.01, Figures 5(g)–5(j)). In the CSF and blood at different periods, only levels of Prx2 in the CSF within 5-7 days of onset had predictive significance for prognosis.

### 3.6. Relationship between Clinical Scores and the Ratio of Prx2 in the CSF and the Blood

We assessed the relationship between clinical scores and the ratios of Prx2 in the CSF and the blood at different times. We found that 14 patients had both CSF and blood within 3 days of the onset, and the ratio of Prx2 during this period was positively correlated with the admission Hunt-Hess score (*R* = 0.586, *P* < 0.05, Figure 3(e)) and negatively correlated with the GOS (*R* = ‐0.605, *P* < 0.05, Figure 5(e)). In Figure 5(k), we evaluated its predictive power for poor prognosis, with an AUC of 0.9167, but *P* value of 0.0679. Twenty-eight patients simultaneously had CSF and blood within 5-7 days. The ratio of Prx2 during this period was not associated with the admission Hunt-Hess score (Figure 3(f)) or the GOS (Figure 5(f)) and showed no predictive power for poor prognosis (AUC = 0.5739, *P* > 0.05, Figure 5(l)).

## 4. Discussion

The essence of Prx2 is a peroxidase encoded by the Prdx2 gene [14], which has the effect of decomposing reactive oxygen species (ROS) [15]. In our earlier study, we determined the protective effect of Prx2 in animal experiments. In the mouse CNS, Prx2 is mainly expressed in the neurons. 6 h after experimental SAH, the expression of Prx2 in the mouse brain tissue increased significantly, reached its peak at 12 h, and returned to the baseline level after 7 days. Injection of the functional inhibitor of Prx2, adenanthin [16], into the lateral ventricle of the mice significantly increased the content of malondialdehyde (MDA) in the brain tissue after SAH [8]. Prx2 is abundant in red blood cells [17]. We found that the lytic erythrocytes and damaged neurons can release Prx2, which may act as damage-associated molecular patterns (DAMPs) in the microenvironment to activate the microglia, promote the release of inflammatory factors from the microglia, and cause neuronal apoptosis [9].

Although a series of animal experiments have investigated the role of Prx2 in brain injury-related diseases [18–20], few studies have tracked the changes in the Prx2 levels in patients. Prx2 is one of the most significantly elevated proteins in the CSF after SAH [7]. We observed the changes in the Prx2 levels at different stages of onset and correlated them with the clinical conditions. We found that the levels of Prx2 in the CSF were significantly increased in the acute phase (0-3 days) and correlated with the severity of the patient's onset. The severity of a patient's disease is often related to the amount of bleeding or the degree of brain tissue damage. At this stage, the higher the Hunt-Hess score, the more lysed red blood cells or damaged neurons, the more Prx2 is released, and higher levels of Prx2 in the CSF were observed. In the process of disease progression, patients in poor condition are more prone to CVS after cerebral ischemia, hypoxia, and metabolic disorders. We found that the patients with CVS had relatively higher levels of Prx2 in the CSF within 5-7 days, while there was no difference in the Prx2 levels in the CSF within 3 days. Acute neuronal damage occurred 3 days after onset, and relatively more red blood cells ruptured during this period. During the 5- to 7-day period, although the levels of Prx2 were still high, it may be caused by secondary damage to the neurons, which may be caused by various metabolic wastes in the microenvironment. Prx2 levels during this period were indicative of the patient's clinical condition. In addition, we also found that the Prx2 levels in the CSF within 5-7 days and 3 days have good and no predictive significance for the prognosis, respectively. This also suggests that the levels of Prx2 in the CSF within 3 days mainly reflect the severity of the disease, while the levels of Prx2 in the CSF on days 5-7 reflect the clinical conditions of the patient. Regarding changes in the Prx2 levels in the CSF, we hypothesized that compared with the acute phase, patients whose Prx2 levels in the CSF can be resolved early within 5-7 days have a better prognosis. But only 14 patients had CSF within 0-3 days and 5-7 days at the same time. We did not have enough data to generate and test our hypothesis. We will expand the patient sample size and test this hypothesis in the next trial.

In clinical diagnosis and treatment, blood is easier to obtain than CSF and has greater value as a clinical marker. Unfortunately, although the levels of Prx2 in the blood continued to decrease after the onset of the disease, we did not find a correlation between it and the clinical condition of the patient. Decreased levels of Prx2 in the blood may be due to stress responses, adverse systemic conditions after the onset, or suggest a diminished capacity to regulate oxidative stress. In this study, we mainly focused on the changes in the patient's nervous system. The levels of Prx2 in the blood may be related to some complications involving other systems, such as cardiac dysfunction or pulmonary edema [21]. If we need to observe the clinical significance of changes in Prx2 levels in the blood, we may need to expand the scope of our research and pay attention to the patient's general condition.

The levels of Prx2 in the CSF can reflect the state of the CNS, and those in the blood can reflect the systemic state or the ability to regulate oxidative stress. Therefore, we speculated that the ratio of Prx2 in the CSF and the blood could also be used as a marker, so we observed the correlation between clinical scores and the ratio of Prx2 in different periods. To our surprise, the ratio of Prx2 within 3 days of onset was positively correlated with the admission Hunt-Hess score and negatively correlated with the GOS at 3 months after the discharge, which meant that during this period, the higher the level of Prx2 in the CSF and the lower the level of Prx2 in the blood, the more severe the patient's disease and the poorer the prognosis. High levels of Prx2 in the CSF mean severe damage to the CNS, while low levels of Prx2 in the blood may mean poor self-regulation. This was the only indicator associated with both the onset and the prognosis. However, this indicator had no predictive power for poor prognosis, which may be because only 2 of the 14 patients had a poor prognosis, resulting in nonsignificant data. The ratio of Prx2 within 5-7 days had no clinical significance, which may be because the disease had entered a compensatory phase and the patient gradually recovered, resulting in a decline in the clinical significance of this ratio.

In this study, we only observed changes in the Prx2 levels in the clinical samples within 0-3 days and 5-7 days after the onset of the disease. During this period, the Prx2 levels continued to rise or fall, and we did not observe a recovery of the Prx2 levels. As the most changed protein in CSF [7], Prx2 response to the disease may also be more sensitive. Thus, is it possible that when Prx2 returns to the normal level, it could mean an improvement in the patient's condition or even serve as an indicator of the patient's recovery? This will also be the focus of our next work, and we will extend the observation period in future work to better understand the role played by Prx2. Due to the lack of sufficient sample size, Prx2 levels in the blood within days and the ratio of Prx2 in the CSF and blood within 3 days had poor predictive power for poor prognosis. Despite the large AUC, the *P* value was greater than 0.05. In the next experiment, we need to further expand the sample size to observe the potential predictive value of these two indicators for poor prognosis.

## 5. Conclusions

After the onset, the level of Prx2 in the CSF and the blood increased and decreased, respectively. Levels of Prx2 in the CSF and the ratio of Prx2 in the CSF and the blood within 3 days of onset can be used as a biomarker to detect the severity of the disease and the clinical status of the patient.

## Figures and Tables

**Table 1:** Patient characteristics.

Variable	Value
Patients	
Males	19
Females	15
Age	55.7 ± 9.0

Hunt-Hess	
I	11
II	12
III	5
IV	6
V	0
CVS	7

GOS	
1	0
2	1
3	5
4	20
5	8

## Data Availability

The data supporting the results have been provided in the article.
